# Novel 3-D Macrophage Spheroid Model Reveals Reciprocal Regulation of Immunomechanical Stress and Mechano-Immunological Response

**DOI:** 10.1007/s12195-024-00824-z

**Published:** 2024-10-14

**Authors:** Alice Burchett, Saeed Siri, Jun Li, Xin Lu, Meenal Datta

**Affiliations:** 1 Department of Aerospace and Mechanical Engineering, University of Notre Dame, Notre Dame, IN USA; 2 Department of Applied and Computational Mathematics and Statistics, University of Notre Dame, Notre Dame, IN USA; 3 Department of Biological Sciences, University of Notre Dame, Notre Dame, IN USA

**Keywords:** Solid stress, Inflammation, Microenvironment, Myeloid cells, Polarization

## Abstract

**Purpose:**

In many diseases, an overabundance of macrophages contributes to adverse outcomes. While numerous studies have compared macrophage phenotype after mechanical stimulation or with varying local stiffness, it is unclear if and how macrophages directly contribute to mechanical forces in their microenvironment.

**Methods:**

Raw 264.7 murine macrophages were embedded in a confining agarose gel, and proliferated to form spheroids over days/weeks. Gels were synthesized at various concentrations to tune stiffness and were shown to support cell viability and spheroid growth. These cell-agarose constructs were treated with media supplements to promote macrophage polarization. Spheroid geometries were used to computationally model the strain generated in the agarose by macrophage spheroid growth. Agarose-embedded macrophages were analyzed for viability, spheroid size, stress generation, and gene expression.

**Results:**

Macrophages form spheroids and generate growth-induced mechanical forces (i.e., solid stress) within confining agarose gels, which can be maintained for at least 16 days in culture. Increasing agarose concentration increases gel stiffness, restricts spheroid expansion, limits gel deformation, and causes a decrease in Ki67 expression. Lipopolysaccharide (LPS) stimulation increases spheroid growth, though this effect is reversed with the addition of IFNγ. The mechanosensitive ion channels Piezo1 and TRPV4 have reduced expression with increased stiffness, externally applied compression, LPS stimulation, and M1-like polarization.

**Conclusions:**

Macrophages alone both respond to and generate solid stress. Understanding how macrophage generation of growth-induced solid stress responds to different environmental conditions will help to inform treatment strategies for the plethora of diseases that involve macrophage accumulation and inflammation.

**Supplementary Information:**

The online version contains supplementary material available at 10.1007/s12195-024-00824-z.

## Introduction

From atherosclerotic plaques to tuberculosis granulomas to solid malignant tumors, macrophages play important roles in immune effector function and orchestration in aberrant tissue masses, and as active constituents of the mechanical microenvironment [1]. As innate immune cells, they are not only part of the first line of defense against pathogens, but they also contribute to tissue repair and help coordinate the broader immune response. Macrophage phenotype is highly plastic and ebbs between pro- and anti-inflammatory states, as these cells sense and correspondingly respond to diverse and dynamic microenvironments [2]. Macrophages present in tissues are either resident or derived from circulating monocytes that differentiate into macrophages upon vascular extravasation and tissue infiltration [3]. During an inflammatory response, an injured or diseased site will accumulate macrophages, both through the recruitment of circulating monocytes and local proliferation of bone marrow and embryonic-derived macrophages [4]. For example, upon tissue injury, vascular endothelial cells upregulate adhesion molecules that allow patrolling monocytes to adhere to the vessel wall, where they withstand shear stress from blood flow, and eventually enter the tissue between endothelial junctions [5].

While macrophage phenotype and function, known also as polarization, is traditionally thought to exist as either pro-inflammatory (“M1-like”) or anti-inflammatory (“M2-like”), their dynamic cell state can lie on a continuum between inflammation-accelerating and inflammation-inhibiting responses [6]. Macrophages adopt and shift between these polarizations in any tissue; generally, equilibrium between the two ends of the spectrum is essential for homeostatic tissue maintenance and repair. However, an over- or under-active macrophage response can disrupt this tenuous balance, particularly in cases where macrophages accumulate in large quantities.

Elevated macrophage populations in diseased tissue are often correlated with worse prognosis, particularly in diseases where altered tissue mechanics contribute to the pathophysiology [7]. Cancer, wound healing, bacterial infections, and other disease settings involve altered tissue mechanics, which impact immune surveillance and response [8]. For example, atherosclerotic plaques physically disrupt blood flow, and their mechanical stability determines if they will rupture, leading to downstream ischemic events [9]. Macrophages accumulate in plaques, where they contribute to this mechanical instability, increasing the risk of life-threatening events such as a stroke. Macrophages infiltrate tumor microenvironments in high numbers, with the density of tumor-associated macrophages correlating with worse prognoses in cancers including glioblastoma, ovarian cancer, and breast cancer [10–14]. In glioblastoma, macrophages can comprise up to 50% of the tumor bulk, promoting tumor progression and treatment resistance [15–17]. Macrophages also contribute to increased collagen deposition in hypertrophic scars and heart attack scarring, leading to host tissue damage and diminished function [18, 19].

In addition to classical biochemical cues, macrophages also respond to mechanical stimuli such as shear stress, tissue viscoelasticity, cyclic compression or stretching, and hydrodynamic pressure changes [20]. This response has been characterized previously in the context of the cardiovascular and skeletomuscular systems [21–24]. Macrophages experience a wide range of tissue mechanical properties, with Young’s moduli on the order of single kilopascals in the brain to tens of gigapascals in bone [25, 26]. In vitro experiments show that macrophages have a stronger inflammatory response when cultured on stiffer 2-D substrates [27–31]. However, the opposite effect is observed when cells are cultured in a 3-D matrix. Macrophages in stiffer matrices in vitro and in vivo have a more immunosuppressive, M2-like phenotype [32–34]. Thus, a physiologically relevant *in vitro* model is essential to understanding how macrophages respond—and contribute—to mechanical stimuli in the body.

Here, we aimed to characterize the mechanical deformation that macrophages generate through 3-D growth in a confining agarose gel, simulating the mechanics of the tissue microenvironment independent of confounding biochemical cues or matrix degradation. Macrophages (RAW264.7) in single-cell suspension were embedded in agarose gels of varying substrate concentrations to span a range of physiologically relevant stiffnesses. As the agarose-embedded cells proliferated to form spheroids, they displaced the surrounding gel, similar to physiological macrophages en masse (e.g., in plaques, traumatic brain injuries, granulomas, cancerous tumors) generating and exerting growth-induced solid stress on the surrounding tissue [35]. Spheroids in softer gels grew to much larger sizes and caused larger total displacements of the surrounding gel compared to spheroids in stiffer gels. Pro-inflammatory stimulation with LPS also led to an increase in spheroid size, though this effect was reversed with the addition of interferon-γ (IFNγ). The mechanosensitive ion channels Piezo1 and transient receptor potential vanilloid 4 (TRPV4) both decreased in expression with increased stiffness and externally applied compression. Markers of both pro- and anti-inflammatory functional states increased with stiffness and compression. Overall, this work highlights a novel, tunable, and high throughput method of interrogating macrophage immunomechanics and mechano-immunology, with relevance to a wide range of diseases.

## Materials and Methods

### Cell Culture, Gel Formation, and Macrophage Activation/Polarization

RAW264.7 murine macrophages were purchased from ATCC (TIB-71). They were grown in a complete culture medium consisting of DMEM (Corning, 10-013-CV) supplemented with 10% Fetal Bovine Serum (Gibco, 26140079) and 1% penicillin-streptomycin (Corning, 30-002-CI). They were maintained in a humidified incubator at 37 °C with 5% CO_2_. Cells were grown as adherent monolayers and passaged using a cell scraper for mechanical detachment.

To create agarose-embedded 3-D cultures, single-cell suspensions were mixed with low-gelling temperature agarose (Sigma-Aldrich, A0701-25G). First, a 4% agarose solution was made in a complete culture medium and heated in a microwave until dissolved. The liquid agarose was maintained at 48 °C until use. Suspensions of RAW264.7 cells in medium were mixed with a proportional amount of the 4% agarose to create gels with a final concentration of 0.5%, 1%, or 2% agarose and 1000 cells/ml to 10,000,000 cells/ml, depending on the experiment. The cell-agarose solution was pipetted into 2 mm-deep cylindrical molds and left at room temperature to set for 10 min. The gels were then removed from the molds, submerged in complete culture medium with or without supplementation listed below, and maintained under standard culture conditions. The cylindrical molds were made using custom 3D-printed polylactic acid (PLA) rings adhered to a glass coverslip. These were sterilized with 70% ethanol and 30 min of UV light exposure in a biosafety cabinet prior to use.

The gels in each condition were treated immediately after the gel solidified so that the embedded cells experienced the various treatments for the entire time they were embedded in agarose. For all experimental conditions other than variable agarose concentration, macrophages were seeded in 1% agarose gels as described above, and agents were added to the culture media. To simulate bacterial activation of macrophages, the medium was supplemented with 200 ng/ml lipopolysaccharide (LPS, Santa Cruz Biotechnology, sc-3535). For M1-polarization-treated gels, the medium was supplemented with 20 ng/ml IFN-γ (BioLegend, 575302) and 200 ng/ml LPS. For M2-polarization-treated gels, the medium was supplemented with 20 ng/ml IL-4 (Pepro-Tech, 214-14). The volume of the gel was included in the total solution volume to achieve accurate final concentrations. Gels cultured under hypoxic conditions were placed in a Tri-Gas hypoxia incubator with 5% CO_2_ and 1% O_2_. Compressed gels were placed on a 0.4 μm pore size transwell cell culture insert (CellQART, 9310402) and a 3-D printed PLA weight was placed on top to apply 0.14 kPa of compression to the gel to simulate the solid stress measured in murine glioblastoma models [17, 36, 37]. The pores in the transwell membrane enable media exchange with media in the lower well, without allowing the transmigration of cells. Because compression results in anisotropic spheroid growth, and because our size analysis methods rely on 2D projections, we omitted the compressed gels from our size analysis to avoid misrepresenting the actual spheroid size due to a potential flattening effect.

Depending on initial cell density, these gels could be maintained for several days to several weeks before they became overgrown. Different seeding densities and culture times (Fig. S1) were chosen based on requirements for cell number spheroid size, and biological material needed for each assay.

### Viability and Metabolic Activity Assays

For live/dead staining, the gels were incubated with 2 μg/ml Calcein-AM (BioLegend, 425201) and 1 μg/ml Propidium Iodide (PI, MP Biomedicals, 0219545810) in complete medium for 30 min at 37 °C. 0.5%, 1%, and 2% agarose gels (100,000 cells/ml starting density on day 5 or else 1000 cells/ml at day 16) were imaged on a point-scanning confocal microscope (Nikon AXR) to obtain representative spheroid images. Representative images were shown as a maximum-intensity projection of an image stack. For the viability versus agarose concentration analysis, cell-laden gels were created in a 96-well plate in 0.5%-2% agarose at an initial seeding density of 10,000 cells/ml so that individual cells could be easily distinguished. After 1 day in culture, three wells from each condition were imaged 1 day after seeding at 4x magnification in stacks with 100 µm step size and 500 µm total depth (Nikon Ti2). Each stack was compressed into a maximum intensity Z projection and processed in ImageJ. For both the red and green channels, the background was subtracted using the Subtract Background function with a radius of 500 pixels. The intensity for the green and red channels was set as a uniform threshold across the image dataset, and the Analyze Particles function was used to count the number of green (live) and red (dead) cells. The viability is calculated as the number of live cells divided by the total (live plus dead) cells.

For the metabolic (3-(4, 5-dimethylthiazolyl-2)− 2,5-diphenyltetrazolium bromide (MTT) assay, 50,000 cells were seeded in 50 µl of agarose, with 50 µl of media added on top. At each time point, the 100 µl of MTT reagent (ATCC, 30-1010 K) was added to each well and incubated for 2.5 h. The detergent reagent was added and incubated overnight to ensure complete dissolution of the agarose gel. The plate was read on a Spark multimode plate reader (Tecan) at 570 nm with a reference wavelength of 670 nm. Blank values for each agarose concentration were obtained from cell-free gels and subtracted from the readings to obtain the final absorbance values.

### Whole Gel Spheroid Imaging

Gels were seeded with 100,000 cells/ml and cultured for 3 days under the various conditions described above. Representative images (5-9) of each condition were taken at 10x magnification using a phase-contrast microscope, resulting in between 26 and 87 spheroids measured per condition (Leica DMi1). Spheroid outlines were manually created for each image, and the area of identified spheroids was quantified using ImageJ’s Analyze Particles function. For representative images, images were cropped, converted to black and white, and the sharpness was set to 100% in Microsoft PowerPoint.

For the red actin-stained spheroids, 100,000 cells/ml gels at day 4 were fixed overnight in 4% paraformaldehyde in PBS (Thermo Fisher, J19943.K2) at 4 °C, then rinsed with PBS and incubated with CellMask™ Orange Actin Tracking Stain (1:1000, Thermo Fisher, A57244) for 48 h at 4 °C. Representative image stacks were acquired using a multiphoton microscope (Leica Stellaris 8 DIVE).

### PCR

Gels intended for PCR were seeded at a density of 10,000,000 cells/ml to ensure that enough RNA could be extracted from a small volume of agarose that was compatible with the extraction kit. RNA was isolated after 2 days of culture to avoid overgrowth and disparity in RNA quantity between conditions which encourage differential proliferation rates. For each sample, a small piece of gel (~ 100 μl volume) was dissolved in 400 μl TRI-reagent (Zymo Research, R2050-1-200) and the RNA was purified using an RNA isolation kit (Zymo Research, R2051). Gene expression was analyzed using TaqMan primers for Arg1 (Thermo Fisher Scientific, Mm00475988_m1), Nos2 (Mm00440502_m1), Piezo1 (Mm01241549_m1), TRPV4 (Mm00499025_m1), Ki67 (Mm01278617_m1), Caspase 3 (Mm01195085_m1), and GAPDH (Mm99999915_g1). The raw PCR data was analyzed using qPCR Design and Analysis app (Thermo Fisher Scientific). This program selects one sample in the control group (1% agarose) to which every other sample is normalized. Gene expression was normalized to GAPDH and then normalized to one control sample and reported as 2^−ΔΔCt^. Each condition and gene had between 3 and 6 biological replicates. Data points that were clear outliers (e.g. due to pipetting errors) and that were determined by the Grubbs outlier test to be outliers were removed.

### Unconfined Compression Testing

The compressive moduli of the agarose gels were obtained using an ElectroForce 5500 (TA Instruments) mechanical testing instrument fitted with a 5N load cell for unconfined compression testing. Low melting point agarose gels were made as described above and cast into 2 mm thick disks. Using a 6 mm diameter biopsy punch, smaller cylindrical samples were created. These were equilibrated at 37 °C in media in the same incubator used for cell culture for the duration indicated. After being initially aligned with the gel, the upper platen of a 5N load cell was programmed to descend at a rate of 0.0075 mm/s for a total of 1 mm (50%) deformation. This method was used to test three to six gels of 0.5%, 1%, and 2% agarose. The Young’s modulus of each sample was determined by finding the slope of the linear region of the stress-strain curve. Data from each sample was cropped to include only the approximately linear region and an average value from 3 or 4 samples was taken to be the Young’s modulus at each agarose concentration.

### Computational Modeling

To investigate the distribution of deformation around the spheroids, a computational modeling approach was employed using COMSOL Multiphysics. Agarose gels with concentrations of 0.5%, 1%, and 2% were modeled as linear elastic materials (E = 2000, 19830, and 99596 Pa respectively) [38]. The spheroid geometry was obtained through image processing techniques and subsequently implemented in COMSOL. The initial geometry of the spheroid was considered as a sphere with a 5-micrometer radius, mimicking the size of a single cell from which the spheroid grows. We considered the macrophage spheroid as an object of uniform stiffness for simplicity. The final geometry was considered as a circle or oval approximately matching the dimensions of spheroids based on fluorescent actin images taken at day 4. Because we employed displacement-driven boundary conditions in our simulations, the exact mechanical properties (e.g., Young’s modulus) of the spheroid were not required. Through the manual application of prescribed displacement boundary conditions, the spheroids were expanded from the initial to final geometry within the agarose gel. This approach allowed for the exploration of the displacement distribution around the spheroids. The modeling simulations were conducted in 2-D, providing a comprehensive analysis of the deformation distribution within the agarose gel environment. This methodological approach enables a detailed examination of the impact of agarose gel concentration on the mechanical behavior of macrophage spheroids in a controlled and reproducible manner.

### Statistical Analysis

Statistical analyses and data visualization were done using GraphPad Prism and Excel. For cell viability, each comparison was made using the Mann-Whitney test. For MTT data, comparisons were made using a 2-way analysis of variance (ANOVA). For spheroid size, groups were compared using the Kruskal-Wallace one-way ANOVA, followed by Dunn’s multiple comparisons test. PCR data was compared using the Mann-Whitney test. Error bars represent standard error of the mean (SEM) and asterisks indicate statistical significance (*p < 0.05, **p < 0.01, ***p < 0.001, ****p < 0.0001).

## Results

### Agarose-Embedded Macrophages form Spheroids with Long-Term Viability

To determine whether macrophages alone can generate solid stress, we utilized an agarose hydrogel to serve as a confining gel. As a plant-derived material, agarose is biologically inert to mammalian cells, and is also physically and chemically stable, with suitable biocompatibility [39, 40]. The mechanical properties of agarose are also easily tunable, as Young’s modulus increases exponentially with molar concentration [38]. We measured the stiffness of 0.5%, 1%, and 2% low melting point cell culture grade agarose using an unconfined compression testing device to obtain stress-strain curves (Fig. S2a). From these, we calculated the average Young’s modulus at each concentration. After 1 day of equilibration, 0.5%, 1%, and 2% agarose had average Young’s moduli of 3.8 kPa, 12.1 kPa, and 44.8 kPa, respectively. These approximately correspond to the stiffness ranges measured in the brain, healthy heart, and fibrotic scar tissue, respectively [26, 41].We tested gels that had been equilibrated in media under standard cell culture conditions for either 1 day or 17 days, finding no obvious change in material properties over time (Fig. S2b).

**Fig. 1:**
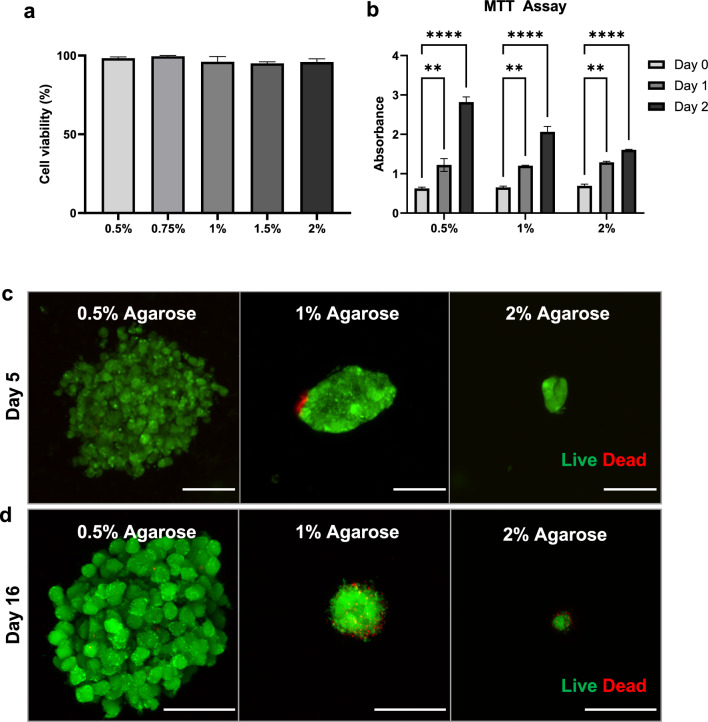
Macrophage spheroids embedded in agarose form aggregates with sustained viability. **a** Percent of live cells one day after embedding in agarose gels of varying concentrations. There was no significant difference between any groups, and each group had n = 3. **b** MTT assay results for cells embedded in agarose at varying concentrations at three time points (n = 3). **c** Representative images of spheroids at day 5, stained with calcein-AM (green) for live cells, and propidium iodide (red) for dead cells. Scale bar is 50 µm. **d** Representative images at day 16 stained for live (green) and dead (red) cells. Scale bar is 50 µm. Error bars represent SEM and asterisks indicate statistical significance (*p < 0.05, **p < 0.01, ***p < 0.001, ****p < 0.0001)

Having determined that agarose is mechanically suitable for disease modeling, we next verified that it was biocompatible with our RAW264.7 murine macrophage cell line. Because the agarose embedding process involved mixing live cells with warm molten agarose, we tested whether the embedding process would result in cell death. Macrophages in single-cell suspension were mixed with agarose to create a uniform gel with individual cells uniformly dispersed throughout. We found that macrophages embedded in agarose between the concentrations of 0.5% and 2% had an average viability ranging from 95.1% to 99.5%, with no significant difference between conditions, as measured 1 day after embedding via calcein-AM and PI staining (Fig. 1a). The metabolic activity of agarose-embedded cells measured in an MTT assay increased significantly over time in all three agarose concentrations (Fig. 1b). Metabolic activity was uniform between 0.5%, 1%, and 2% agarose initially after embedding and 1e day after embedding. By day 2, however, cell proliferation increased total metabolic activity per gel, with softer gels having a larger increase in activity compared to stiffer gels.

Over time, the agarose-embedded macrophages proliferated to form “spheroids,” or clumps of multiple cells that expand in place (Fig. 1c). We intentionally chose a material that the cells could not manipulate, as we sought to assess the ability of macrophages to generate growth-induced solid stress through expansion. Any cell proliferation must therefore cause solid stress, as the cells must displace the elastic material to make room for the growing 3D structure. Agarose-embedded macrophage spheroids displayed excellent long-term viability, with healthy spheroids observed 16 days after seeding (Fig. 1d). For longer-term culture, cells were seeded at a lower density, and as a result grew more slowly, resulting in smaller spheroids observed on day 16 compared to larger spheroids observed at earlier timepoints when seeded at a higher density. Live spheroid constructs can likely be maintained for much longer periods than reported here, as we observed the time in culture to be limited only by the eventual overgrowth of the gel by cells.

### Macrophages Generate Solid Stress by Displacing the Confining Agarose Gel

**Fig. 2:**
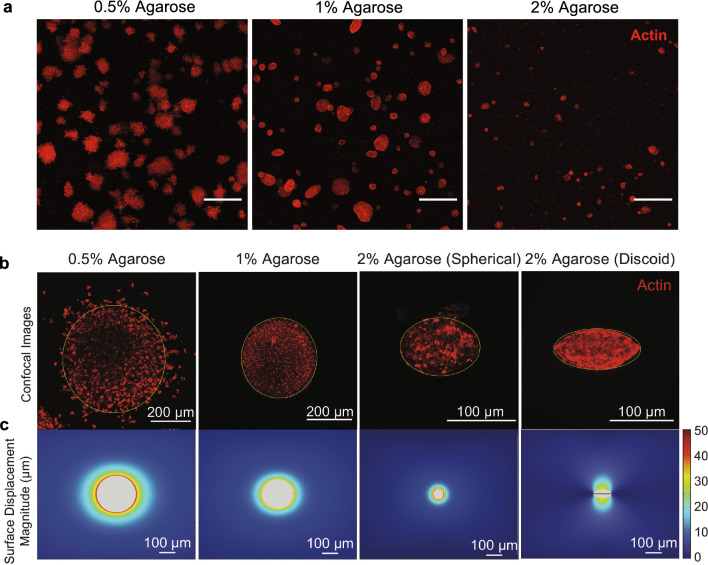
Agarose concentration modulates gel stiffness, mechanical interactions, and spheroid expansion. **a** Representative maximum intensity projections of actin-stained spheroids (red) in 0.5%, 1%, and 2% agarose at day 4. Scale bar is 200 µm. **b** Representative images of single actin-stained spheroids to be used to inform computational models. **c** Representative COMSOL models of the strain field generated from each spheroid as it expands into a viscoelastic medium

The 3D morphology of the spheroids was captured via confocal imaging of actin-stained spheroids after 4 days of culture. This reveals a variety of shapes, not only spheroidal aggregates (Fig. 2a). In the softer 0.5% gel, individual cell profiles were visible as they protruded into the surrounding gel, while stiffer gels tended to result in smoother spheroid edges (Fig. 1c, d). Due to the computational complexity of modeling an exact 3D morphology, we completed our deformation modeling using 2D maximum intensity projections of representative spheroids stained for actin (Fig. 2b).

Given an estimate of the spheroid geometry and the mechanical properties of the gel, we generated a simulation of the displacement field in the gel surrounding the spheroids using COMSOL (Fig. 2c). Simulations of spheroid displacement during growth are shown based on the corresponding fluorescent images. The color scale indicating displacement magnitude (μm) was adjusted for clear representation of the entire displacement field rather than maximum displacement. In Fig. 2c from left to right, the first three simulations for a spheroid embedded in 0.5%, 1%, and 2% agarose gels assume no microcrack developments in the gels and an initial uniformly spherical geometry with a 5 μm radius, while the simulation for the spheroid in the 2% agarose gel with a discoid shape assumes formation and propagation of a microcrack in the gel during initial spheroid growth, as observed in similar agarose-embedding studies [42]. While we did not directly observe crack formation, we applied the model to determine the displacement field in the case of a crack formation. The greatest solid stress-induced deformations are observed in 0.5% gels (with maximum gel displacements of ~ 250 μm from the initial assumed diameter of 10 μm [single-cell spherical geometry]), compared to stiffer 2% gels with spherical (40 μm) and discoid (42 μm) shapes. The extent of deformation relative to spheroid size propagates further into the surrounding matrix with increasing gel concentrations.

These data demonstrate that increasing the rigidity of the surrounding agarose matrix by elevating gel concentration significantly restricts spheroid expansion and modulates growth morphology. The decreased gel displacement and altered shape in stiffer gels indicate that the increased mechanical resistance of the matrix impedes outward growth. In contrast, the more mechanically permissive and compliant 0.5% agarose gel allows for greater spheroid expansion which maintains a rounded shape. Solid stress generation can be accurately inferred from deformation of the surrounding elastic material.

### Spheroid Size Decreases with Increasing Agarose Concentration and Increases with LPS Stimulation

**Fig. 3:**
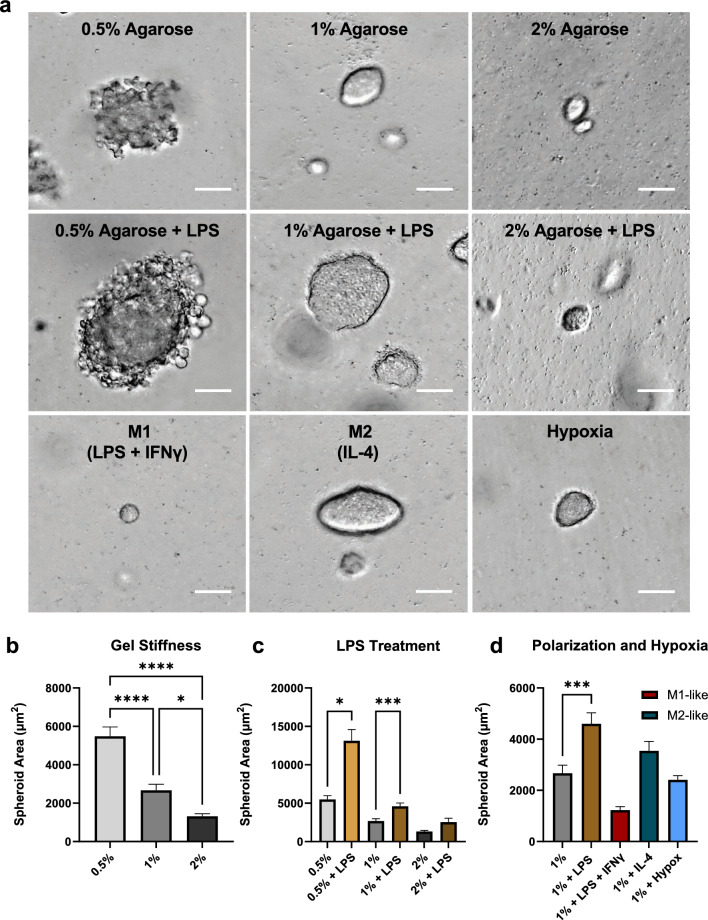
Spheroid size varies with agarose stiffness and macrophage stimulation/polarization** a** Phase-contrast images of representative day 3 spheroids used for size quantification, shown under various culture conditions. Scale bar is 50 µm. **b** Spheroid area as measured from phase-contrast images for 0.5%, 1%, and 2% agarose. **c** Spheroid area comparison between different agarose concentrations with and without the addition of LPS. **d** Spheroid area comparison between regular 1% agarose cultures, and 1% agarose cultures with the addition of M1-like polarizing agents, M2-like polarizing agents, LPS, and hypoxic culture conditions. Between 26 and 87 spheroids were measured in each condition, error bars represent SEM and asterisks indicate statistical significance (*p < 0.05, **p < 0.01, ***p < 0.001, ****p < 0.0001)

After establishing that agarose can support healthy spheroid culture and computationally predicting the resulting displacement field, we next explored the effects of various biologically relevant stimuli on spheroid size (and therefore stress generation). In addition to the clearly visible effects of agarose concentration (Fig. 2a), chemical stimuli added to the culture media also influenced spheroid size. The addition of 200 ng/ml lipopolysaccharide (LPS) serves to simulate a bacterial infection, as it is derived from a common bacterial fragment and is known to elicit potent macrophage responses [43]. To induce the canonical pro-inflammatory M1-like polarization of macrophages (denoted “M1” in the figure), the cultures were treated with 200 ng/ml LPS plus 20 ng/ml interferon-γ (IFNγ) [44]. For canonical anti-inflammatory M2-like polarization (denoted “M2”), cells were treated with 20 ng/ml interleukin-4 (IL-4) [45]. The spheroids were grown for 3 days before phase-contrast images were taken for size analysis. Images were taken at random locations within the gel, and each contained several spheroids. Aggregates larger than singlet or doublet cells created an easily visible outline in phase-contrast images and their sizes were compared based on the spheroid area. Representative single spheroids are shown in Fig. 3a. We found that metrics such as solidity and eccentricity (measures of smoothness and elongation, respectively) had no meaningful differences between the tested conditions (data not shown).

Spheroid size was significantly increased in 0.5% agarose (5484 µm^2^) compared to 1% agarose (2669 µm^2^) and was significantly reduced in 2% agarose (1316 µm^2^) compared to 0.5% (Fig. 3b). Thus, spheroid growth appeared to be inhibited with increasing matrix stiffness. Adding LPS increased spheroid size to 13117 µm^2^ in 0.5% agarose and 4598 µm^2^ in 1% agarose, a difference that was statistically significant (p = 0.003, p = 0.03, respectively) (Fig. 3c). As agarose stiffness increased, the effect of LPS diminished, and the change in size observed in 2% agarose with the addition of LPS was not statistically significant (to 2543 µm2, p = 0.56). The addition of IFNγ with LPS (M1-like polarization) completely reversed the effect of LPS alone, causing a statistically significant decrease in size (to 1226 µm^2^ on average) compared to LPS stimulation (p < 0.0001, comparison not shown), and a trending, nonsignificant decrease in size compared to control (Fig. 3d). M2-like polarization caused a non-significant, slight increase in spheroid size to 3544 µm^2^, and resulted in spheroids that were statistically significantly larger than the M1-like treatment group (p = 0.0003, comparison not shown) (Fig. 3d). Spheroid grown in hypoxia averaged 2408 µm^2^, not significantly different in size from control spheroids grown in normoxia.

### Macrophages in 3D Gels Display Altered Mechanical and Inflammatory Gene Expression in Response to Varied Mechano-Chemical Stimuli

**Fig. 4:**
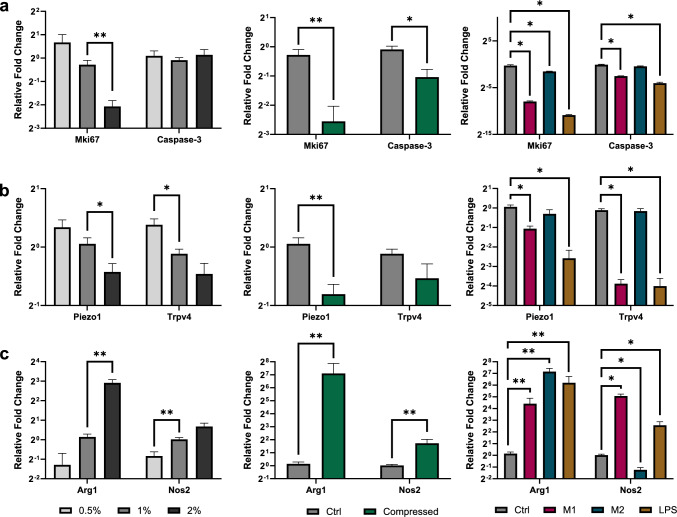
Macrophages show altered gene expression in response to both mechanical and chemical stimuli qPCR analysis of genes related to proliferation and apoptosis (**a**), mechanosensitive ion channels (**b**), and macrophage polarization (**c**). Asterisks represent the p-value (*p < 0.05, **p < 0.01, ***p < 0.001, ****p < 0.0001) from a Mann-Whitney test comparing each condition to the 1% agarose condition (also labeled “Ctrl”), and error bars represent SEM (n = 3–6). Bars without an asterisk notation have no significant difference from the 1% control condition

We next compared the gene expression of macrophages under various mechanical and/or chemical conditions using qPCR. *Mki67* (encoding Ki67) showed a trending decrease with increasing agarose concentration, with the reduction from 1% to 2% being statistically significant (0.23-fold, p = 0.002) (Fig. 4a). This aligns well with the spheroid size data (Fig. 3b), implicating reduced proliferation behind the observed reduced spheroid size in stiffer gels. There was no significant difference in *Casp3* (encoding Caspase−3) expression with agarose concentration. This, together with the near-complete viability observed via calcein-AM and PI staining, indicate that apoptosis is likely not responsible for the reduced size with increased stiffness.

We also tested the effect of externally applied compression, to determine whether compressive stress could impact the macrophages, potentially completing a macrophage-compression feedback loop. We applied 0.14 kPa of uniaxial compression, approximately the magnitude measured in murine glioblastoma models [17, 36, 37]. This resulted in a significant decrease in *Ki67* expression in the compressed gels (0.10-fold), and surprisingly, a concurrent decrease in *Casp3* (0.35-fold) (Fig. 4a). This indicates that a reciprocal regulation may exist between macrophages and the solid stress they generated, though this warrants further investigation. M1-like polarization, M2-like polarization, and LPS stimulation all significantly decreased *Ki67* expression (0.004-fold, 0.35-fold, and 0.0005-fold, respectively). This is interesting, given that the addition of LPS was observed to cause a significant increase in spheroid size, and M2-like polarization caused a trending, non-significant increase in size (Fig. 3d). This suggests that either *Ki67* gene expression is not an accurate reflection of cell proliferation in this setting, or that spheroid size may change independent of proliferation, such as through changes in individual cell sizes.

*Piezo1* and *Trpv4* (encoding Piezo1 and TRPV4, respectively) were also altered based on gel stiffness and chemical cues. *Trpv4* expression increased with 0.5% compared to 1% agarose, and decreased with both M1-like and LPS treatments (0.07 and 0.06-fold, respectively) (Fig. 4b). *Piezo1* expression also significantly decreased with both M1-like and LPS treatment, though to a lesser degree. *Piezo1* also decreased between 1% and 2% agarose, with a trending decrease between 0.5% and 1%*.* Reduced mechanosensor expression may indicate that a decreased sensitivity to confining solid stress may play a role in the increase in observed increase spheroid size between untreated and LPS-treated gels (Fig. 3c). Interestingly, applied compression caused a significant decrease in both *Piezo1* and *Trpv4* (Fig. 4b).

Finally, we quantified canonical markers of pro-inflammatory M1-like macrophage activation (*Nos2*, encoding inducible nitric oxide synthase [iNOS]) and anti-inflammatory M2-like polarization (*Arg1*, encoding arginase 1) (Fig. 4c). We observed a trending increase in *Nos2* and *Arg1* with increased stiffness, with a statistically significant 111-fold increase between 1% and 2% for *Arg1* and a 0.4-fold decrease from 1% to 0.5% for *Nos 2*. Both *Arg1* and *Nos2* increased significantly with externally applied compression (45-fold and 14-fold, respectively). As expected, *Nos2* increased with M1-like and LPS treatment and decreased with M2-like treatment. However, *Arg1* unexpectedly increased with all three stimulation/polarization treatments, potentially indicating an altered phenotypic response to these stimuli in 3-D compared to previously observed 2-D culture results.

## Discussion

This 3-D model of macrophage spheroid formation and the accompanying computational modeling of the resulting displacement field and downstream cellular and molecular biology readouts combine to create a novel platform for investigating the immunomechanics and mechano-immunology of macrophages in varying biochemical and mechanical microenvironments. We chose to use low melting point agarose to leverage its mechanical tunability, chemical stability, and biological inertness [39, 40]. While this model lacks the adhesive qualities of other tissue-mimetic hydrogels, it enables us to isolate specific mechanical phenomena of interest and exclude confounding factors such as cell migration, changes in gel stiffness and structure due to cell activity, etc. As the spheroids grow into large multicellular structures, each cell experiences cell-cell contact. The inner cells experience contact on all sides, as cells *in vivo*. The cells on the spheroid surface experience cell-cell contact on sides not directly in contact with the inert agarose. Each cell therefore experiences more cell-cell contact than cells commonly grown in 2-D monolayers. This model is thus suited to capture basic mechanobiological phenomena.

To the best of our knowledge, this is the first demonstration of successful spheroid formation, long-term viability, and solid stress generation by macrophages alone. The agarose gel constructs are simple to generate, inexpensive, and reproducible, making them attractive for high-throughput applications, such as drug screening. The model is also highly amenable to more complex co-culture or organoid experiments, as any number of cell types and treatments can be incorporated. It could therefore be used to model biomechanics of other diseases, such as within tuberculosis granulomas where macrophages dominate, or tissue regeneration processes that involve mechanosensitive crosstalk between stromal cells and macrophages [46, 47]. This platform is also compatible with standard RNA extraction methods. Additionally, the agarose gels can be processed in the same way as tissues for histological analysis. We are currently optimizing methods for sectioning and staining for both cryopreservation and paraffin-embedding. This will enable us to determine whether there is spatial heterogeneity in the cellular response to mechanical or biochemical cues within each spheroid.

While many aspects of macrophage responses to various forms of mechanical stress have been studied, relatively little has been shown about the reciprocal regulation of macrophages and solid stress [7]. Our simulation results suggest that the mechanical microenvironment can override intrinsic growth programs to control spheroid expansion. Cells that would otherwise proliferate exponentially are progressively restricted from expansion by stiffer gels which represent the range of stiffnesses found in human tissues. Softer gels allow for a greater displacement of the gel around the spheroid compared to higher-stiffness gels (Fig. 2b). However, in stiffer gels, the gel deformation propagates further relative to the size of the spheroid. This work also reveals an interesting 3-D growth pattern, as macrophages often adopt a flattened discoid shape, rather than a spheroidal shape. This could indicate either a physical process, such as the formation of a planar crack in the gel, or a biological process, such as differential proliferation or tip/leader cell migratory behavior in different regions of the spheroid [48]. Further work to characterize the agarose gel surrounding a spheroid and whether cracks form is underway. A limitation of the computational model is that it provides an estimate of the displacement of the agarose adjacent to the spheroid, but not within the spheroid itself. Future work to characterize the mechanical properties of macrophage spheroids would inform computational modeling of stress distribution within the spheroids.

The most potent biochemical cue that increases macrophage spheroid size and stress generation at all tested agarose concentrations is treatment with LPS, an immunostimulatory bacterial fragment used to mimic the effect of bacterial infection [49, 50]. Interestingly, the addition of IFNγ along with LPS, a standard M1-like polarizing regime, reversed the effect of LPS entirely. IFNγ and LPS are generally thought to synergize to induce an M1-like phenotype, but this 3-D stress-generation model appears to have revealed a context in which the two oppose one another [51]. This corroborates findings from a recent publication that found that IFNγ can counteract a subset of the effects of LPS stimulation in periphery human monocytes, underscoring the importance of the interaction of different environmental factors in macrophage function [52]. Further mechanistic studies utilizing this model will help to elucidate the nuances of LPS-stimulated versus canonically M1-like polarized macrophages. Further work (such as additional MTT assays) will also be required to determine why there is a discrepancy between the trends in spheroid size and the trends in *Ki67* expression, as this could indicate interesting alternative proliferation pathways, or potentially other mechanisms of spheroid growth such as increased cell size. Additionally, further analysis of the macrophage polarization response will allow us to more robustly characterize the macrophage polarization response to these stimuli.

Mammalian cells in culture respond to hypoxic conditions within hours of exposure, with some pathways reaching maximum response in the 24-72 h time range [53, 54]. Interestingly, hypoxia did not significantly alter spheroid size (Fig. 3d). Because the spheroids are growing continuously, spheroid size after 3 days under hypoxia is the result of the combined effects of hypoxia on the cells for the entire experiment. The literature is divided on whether hypoxia will promote a more pro-inflammatory or anti-inflammatory macrophage phenotype, with some observing an increase in M1-associated pathways, and another observing an increase in M2-associated pathways [55, 56]. However, macrophage phenotype is now understood not to lie on a simple M1-M2 spectrum, but can take the form of intermediate states, with pathological macrophages often displaying aspects of both ends of the canonical spectrum [57]. Thus, a lack in clear change to spheroid size (and therefore stress generation), does not indicate that hypoxic conditions are not significant in this context. Further investigation, such as spatial and histological analysis required to determine how the effects of confinement and hypoxia may interact.

Two prominent mechanosensory ion channels—Piezo1 and TRPV4—have been investigated previously in macrophage response to mechanical cues [31, 58–60]. These channels are activated with increased membrane tension, causing calcium influx and resulting in the movement of transcription factors into the nucleus and activation of pathways related to macrophage polarization, proliferation, and function [60]. We saw that macrophage TRPV4 expression decreased with increasing 3D stiffness, the opposite of what has been observed for mammary epithelial cells and chondrocytes in 2D settings [61, 62]. We also observed an increase in an M2-like marker with stiffness. Piezo1 expression by macrophages has been shown to increase on stiffer surfaces [63, 64]. However, we observed decreased Piezo1 expression with increased stiffness. Cells in a confining gel such as agarose that does not support cell adhesion may not experience the increased membrane tension that activates Piezo1 [65]. Applying uniaxial compression to the gel resulted in similar gene expression responses as increasing stiffness for all markers except Caspase−3 (Fig. 3a). The macrophages in the spheroids may therefore be sensing reciprocal mechanical forces rather than mechanical properties of their surroundings in the case of varying stiffness of the agarose gel. For example, the force generated by a growing tumor may therefore be as important as tumor-induced matrix stiffening for the macrophage response, two phenomena that have been previously characterized in cancer research with respect to tumor and stromal cells [66].

It may be noted that a limitation of this model is that the agarose gel does not support cell adhesion. While macrophage phenotype can be modulated by integrin interactions with the extracellular matrix in 2-D and 3-D models [67], there are also important integrin-independent mechanosensing mechanisms. Recent work has shown that the mechanisms by which macrophages both sense and generate growth-induced mechanical forces such as solid stress are distinct from other traditionally adhesion-dependent cells [68]. As opposed to adhesive cells such as fibroblasts, macrophages do not only rely on integrin adhesions to respond to extracellular matrix stiffness but rather respond via a distinct pathway involving cytoskeletal dynamics [68]. Rather than “pulling” on the surrounding matrix, they push outwards through cellular protrusions, and the resulting compressive stress transduces information about the environment stiffness, leading to downstream responses. Macrophages also use an adhesion-independent “amoeboid” migration mode, relying on the expansion of the actin network in their leading edge to migrate, rather than producing traction forces via integrin adhesions [69]. This results in forces that act to expand their substrate in the direction of motion, pushing the material both in front and behind, resulting in a net movement forward [70]. In both cases, macrophages demonstrate their ability to both sense and respond to solid stress on a subcellular scale independent of adhesion. Future work with high-resolution imaging of the macrophage cytoskeleton in these 3D constructs may elucidate this effect on the spheroid scale.

Macrophages have increasingly been a subject of interest as targets for disease treatments. A meta-analysis reports more than 25 clinical trials targeting tumor-associated macrophages in a range of different cancer types [71]. In atherosclerosis, reducing macrophage mass within plaques is a promising strategy, as is increasing the numbers of anti-inflammatory macrophages and decreasing pro-inflammatory macrophages in damaged heart tissue after myocardial infarction [18, 72]. Depleting macrophages in models of skin wounding reduces hypertrophic scar formation [19]. As previously shown, targeting either macrophages or solid stress in glioblastoma improves outcomes [73–75]. Our model is particularly relevant to tuberculosis, where 3-D masses of macrophages comprise the core of tuberculosis granulomas, and they are an important therapeutic target for host-directed therapies [46, 76]. These macrophage-dominated granulomas exert solid stress as they expand, which can be reducedovercome pharmacologically [77]. Thus, our model is well-suited for mechanistic studies and high-throughput screening for macrophage and solid stress-targeting therapeutic approaches. Thus, understanding factors that contribute to macrophage expansion or reduction via a novel 3-D system could inform future treatment strategies.

Overall, this work demonstrates a novel platform to study previously unexplored aspects of macrophage mechanics. Because many diseases involve both altered macrophage content and altered mechanics, this model may elucidate new and targetable pathological interactions between macrophages and solid stress. Understanding how macrophages generate stress, and how they respond to external cues under chronic solid stress, will inform strategies to target or reprogram macrophages in the plethora of diseases that involve macrophage accumulation. This platform also has promise for screening macrophage-targeted drugs and is highly tunable to apply to a range of diseases.

## Supplementary Information

Below is the link to the electronic supplementary material.

Supplementary file1 (PDF 260 KB) Experimental timeline The endpoint assays displayed in the main figures were performed at different timepoints, indicated in this timeline

Supplementary file2 (PDF 31 KB) Mechanical testing of agarose gels (a) Representative stress-strain curves of 2%, 1%, and 0.5% agarose samples. (b) Average Young’s moduli of agarose gels at day 1 and day 17 after formation. N=3-4 for each condition

## Data Availability

All data and codes are available upon request to the authors.
