# Supplementary figures and images for: Novel 3-D Macrophage Spheroid Model Reveals Reciprocal Regulation of Immunomechanical Stress and Mechano-Immunological Response
Source: Cell Mol Bioeng. 2024 Oct 14;17(5):329–44. doi: 10.1007/s12195-024-00824-z (PMC11538219; doi:10.1007/s12195-024-00824-z)

## Experimental Timeline

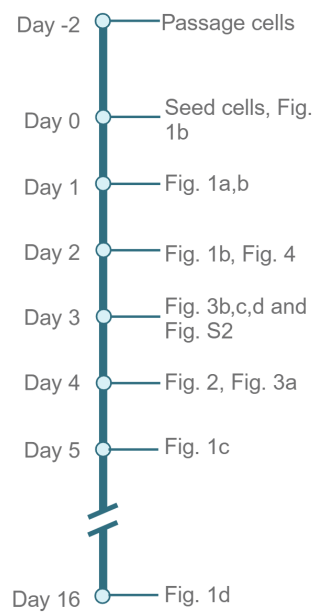

Supplement: Supplementary file 1 — Supplementary file1 (PDF 260 KB) Experimental timeline The endpoint assays displayed in the main figures were performed at different timepoints, indicated in this timeline [file 12195_2024_824_MOESM1_ESM.pdf]

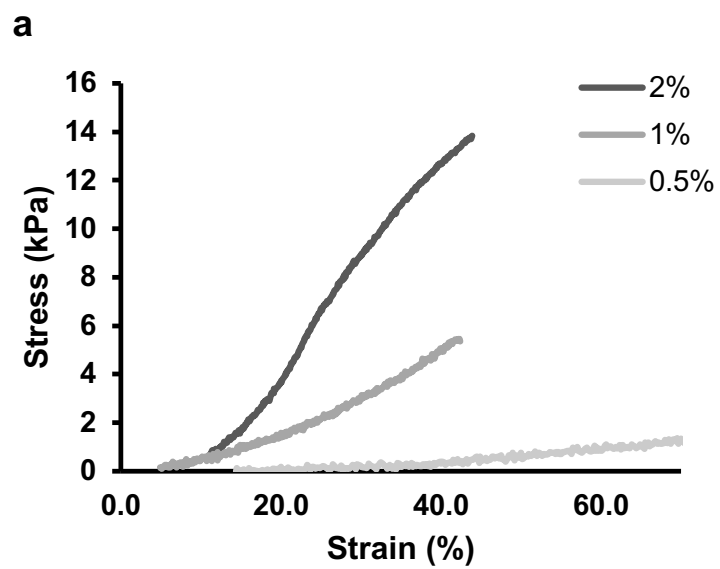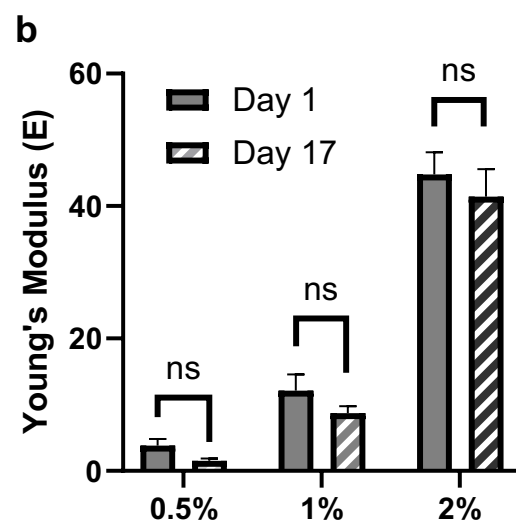

Supplement: Supplementary file 2 — Supplementary file2 (PDF 31 KB) Mechanical testing of agarose gels (a) Representative stress-strain curves of 2%, 1%, and 0.5% agarose samples. (b) Average Young’s moduli of agarose gels at day 1 and day 17 after formation. N=3-4 for each condition [file 12195_2024_824_MOESM2_ESM.pdf]
